# Two Birds with One Stone: Skull Base Meningioma and Jugulotympanic Paragangliomas with Somatostatin Receptor Positron Emission Tomography

**DOI:** 10.3390/diagnostics11091669

**Published:** 2021-09-13

**Authors:** Giorgio Treglia, Mariana Raditchkova, Luca Giovanella, Jean-Jacques Stelmes, Davide G. Bosetti, Francesco Martucci

**Affiliations:** 1 Clinic of Nuclear Medicine, Imaging Institute of Southern Switzerland, Ente Ospedaliero Cantonale, 6500 Bellinzona, Switzerland; mariana.raditchkova-sarnelli@eoc.ch (M.R.); luca.giovanella@eoc.ch (L.G.); 2 Department of Nuclear Medicine and Molecular Imaging, Lausanne University Hospital, 1011 Lausanne, Switzerland; 3 Faculty of Biology and Medicine, University of Lausanne, 1011 Lausanne, Switzerland; 4 Faculty of Biomedical Sciences, Università della Svizzera Italiana, 6900 Lugano, Switzerland; 5 Clinic of Radiation Oncology, Oncology Institute of Southern Switzerland, Ente Ospedaliero Cantonale, 6500 Bellinzona, Switzerland; jean-jacques.stelmes@eoc.ch (J.-J.S.); davidegiovanni.bosetti@eoc.ch (D.G.B.); francesco.martucci@eoc.ch (F.M.)

**Keywords:** PET, positron emission tomography, somatostatin, meningioma, paraganglioma, DOTATATE, gallium-68, MRI, magnetic resonance imaging, skull base

## Abstract

We describe the case of a 74-year-old female patient previously treated with radiation therapy for a meningioma of the skull base and with surgery for a right tympanic paraganglioma. After the morphological progression of the meningioma demonstrated by magnetic resonance imaging (MRI), the patient underwent somatostatin receptor positron emission tomography/computed tomography (SR-PET/CT) with Gallium-68 DOTATATE for restaging. This examination showed increased somatostatin receptor expression by the meningioma and confirmed its extension as already assessed by MRI (endocranial extension, skull base involvement and invasion of the right orbit). Furthermore, SR-PET/CT detected two small right jugulotympanic pararagangliomas with high somatostatin receptor expression. Lastly, SR-PET/CT demonstrated that this patient would be an ideal candidate for peptide receptor radionuclide therapy (PRRT) that can be used for the treatment of progressive/treatment-refractory meningiomas and relapsed paragangliomas with high somatostatin receptors expression, both conditions coexisting in this case.

**Figure 1:**
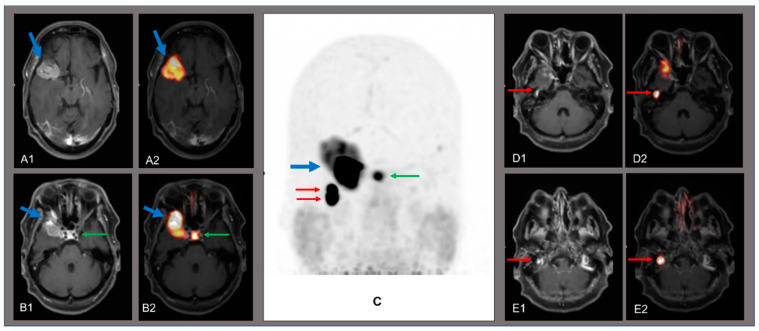
A 74-y.o. female patient previously treated with radiation therapy for a meningioma of the right skull base (16 years before) and with surgery for a right tympanic paraganglioma (10 years before) underwent segmental somatostatin receptor positron emission tomography/computed tomography (SR-PET/CT) for restaging after a progression of the meningioma demonstrated by magnetic resonance imaging (MRI). Segmental SR-PET/CT was performed 60 min after the injection of 146 MBq of Gallium-68 DOTATATE (a radiolabeled somatostatin analogue) and PET images were also fused with the recent MRI images. SR-PET image analysis was performed by using qualitative criteria: areas of increased radiopharmaceutical uptake compared to the background, excluding the sites of physiological radiopharmaceutical uptake, were considered abnormal. Furthermore, semi-quantitative PET image analysis was performed by using the maximal standardized uptake value (SUV_max_). Axial T1-weighted MRI images (**A1**,**B1**,**D1**,**E1**), maximum intensity projection PET image (**C**) and fused PET/MRI images (**A2**,**B2**,**D2**,**E2**) showed several areas of abnormal radiopharmaceutical uptake (blue and red arrows) corresponding to hyperintense lesions at T1-weighted MRI, whereas the pituitary showed physiological tracer uptake (green arrow). In particular, SR-PET revealed areas of increased uptake of radiolabeled somatostatin analogues corresponding to an extensive meningioma of the right skull base (blue arrows in **A1**,**A2**,**B1**,**B2**,**C**). This lesion showed endocranial extension (**A1**,**A2**) and invasion of the right orbit (**B1**,**B2**). The SUV_max_ of this meningioma was 79. SR-PET demonstrated that this meningioma presented a high expression of somatostatin receptors and confirmed the extension of the lesion already assessed by MRI. Interestingly, SR-PET also revealed two areas of increased uptake of radiolabeled somatostatin analogues corresponding to two small right jugulotympanic paragangliomas located in the right jugular foramen and the right middle ear, respectively (red arrows in **C**,**D1**,**D2**,**E1**,**E2**). The SUV_max_ of these paragangliomas was 215. These paragangliomas showed increased expression of somatostatin receptors according to SR-PET findings and only one of them was described in the previous MRI. Several PET radiopharmaceuticals can be used to evaluate brain tumors [1]. In particular, there is currently an increasing interest in the role of SR-PET in patients with meningiomas due to the overexpression of somatostatin receptors by these highly frequent neoplasms [2,3]. Compared to MRI, SR-PET may add additional diagnostic information in patients with meningiomas, particularly in the differential diagnosis of newly diagnosed brain lesions suspicious for meningiomas, delineation of meningioma extent for resection or radiotherapy planning, and differentiation of tumor progression from a post-treatment change [2,3,4,5,6]. Furthermore, SR-PET may be useful to demonstrate the somatostatin receptors expression and to select progressive or treatment-refractory meningiomas which could benefit from peptide receptor radionuclide therapy (PRRT) which is usually a well-tolerated treatment in these patients achieving disease control in most cases [7]. Paragangliomas are neuroendocrine tumors arising from extra-adrenal chromaffine tissues and those located in head and neck region are usually characterized by increased expression of somatostatin receptors and, consequently, they are well-visualized by SR-PET which seems to be the best PET imaging modality for assessing this group of paragangliomas with excellent diagnostic performance [8,9,10]. SR-PET may also be useful to evaluate relapsing or progressive paragangliomas which could benefit from PRRT; this treatment seems promising in paragangliomas expressing high levels of somatostatin receptors with improvement of clinical symptoms and/or disease control [8]. In the described unusual case with concurrent presence of progressive meningioma and relapsed paragangliomas, SR-PET allowed correct evaluation of these tumors based on their increased somatostatin receptors expression. The described patient would be an ideal candidate for PRRT as this treatment could be used in patients with concurrent progressive/treatment-refractory meningiomas and neuroendocrine tumors with high somatostatin receptors expression [11,12], as relapsed paragangliomas in our case.

## Data Availability

The data presented in this study are available on request from the corresponding author.

## References

[1] Treglia G., Muoio B., Trevisi G., Mattoli M.V., Albano D., Bertagna F., Giovanella L. (2019). Diagnostic Performance and Prognostic Value of PET/CT with Different Tracers for Brain Tumors: A Systematic Review of Published Meta-Analyses. Int. J. Mol. Sci..

[2] Unterrainer M., Niyazi M., Tonn J.C., Ilhan H., Bartenstein P., Albert N.L. (2019). Current status of SSR-directed imaging and therapy in meningioma. Clin. Transl. Imaging.

[3] Laudicella R., Albano D., Annunziata S., Calabrò D., Argiroffi G., Abenavoli E., Linguanti F., Vento A., Bruno A., Alongi P. (2019). Theragnostic Use of Radiolabelled Dota-Peptides in Meningioma: From Clinical Demand to Future Applications. Cancers.

[4] Kowalski E.S., Khairnar R., Gryaznov A.A., Kesari V., Koroulakis A., Raghavan P., Chen W., Woodworth G., Mishra M. (2021). 68Ga-DOTATATE PET-CT as a tool for radiation planning and evaluating treatment responses in the clinical management of meningiomas. Radiat. Oncol..

[5] Mahase S.S., O’Brien D.A.R., No D., Roytman M., Skafida M.E., Lin E., Karakatsanis N.A., Osborne J.R., Brandmaier A., Pannullo S.C. (2021). [^68^Ga]-DOTATATE PET/MRI as an adjunct imaging modality for radiation treatment planning of meningiomas. Neuro-Oncol. Adv..

[6] Galldiks N., Albert N.L., Sommerauer M., Grosu A.L., Ganswindt U., Law I., Preusser M., Le Rhun E., Vogelbaum M.A., Zadeh G. (2017). PET imaging in patients with meningioma—Report of the RANO/PET Group. Neuro-Oncology.

[7] Mirian C., Duun-Henriksen A.K., Maier A.D., Pedersen M.M., Jensen L.R., Bashir A., Graillon T., Hrachova M., Bota D., van Essen M. (2020). Somatostatin Receptor–Targeted Radiopeptide Therapy in Treatment-Refractory Meningioma: Individual Patient Data Meta-analysis. J. Nucl. Med..

[8] Taïeb D., Jha A., Treglia G., Pacak K. (2019). Molecular imaging and radionuclide therapy of pheochromocytoma and paraganglioma in the era of genomic characterization of disease subgroups. Endocr.-Relat. Cancer.

[9] Han S., Suh C.H., Woo S., Kim Y.J., Lee J.J. (2018). Performance of 68Ga-DOTA–Conjugated Somatostatin Receptor–Targeting Peptide PET in Detection of Pheochromocytoma and Paraganglioma: A Systematic Review and Metaanalysis. J. Nucl. Med..

[10] Kroiss A.S., Uprimny C., Shulkin B.L., Gruber L., Frech A., Url C., Riechelmann H., Sprinzl G.M., Thomé C., Treglia G. (2019). [^68^Ga]Ga-DOTA-TOC PET/CT in the localization of head and neck paraganglioma compared with [18F]FDOPA PET/CT and [123I]MIBG SPECT/CT. Nucl. Med. Biol..

[11] Basu S., Parghane R.V., Talole S. (2019). Prevalence of hitherto unknown brain meningioma detected on 68Ga-DOTATATE positron-emission tomography/computed tomography in patients with metastatic neuroendocrine tumor and exploring potential of177Lu-DOTATATE peptide receptor radionuclide therapy as single-shot treatment approach targeting both tumors. World J. Nucl. Med..

[12] Assadi M., Rekabpour S.J., Amini A., Dadgar H., Nemati R., Gholamrezanezhad A., Nabipour I., Jafari E., Ahmadzadehfar H. (2021). Peptide Receptor Radionuclide Therapy with ^177^Lu-DOTATATE in a Case of Concurrent Neuroendocrine Tumors and Meningioma: Achieving Two Things in a Single Action. Mol. Imaging Radionucl. Ther..

